# Annexins A2, A6 and Fetuin-A Affect the Process of Mineralization in Vesicles Derived from Human Osteoblastic hFOB 1.19 and Osteosarcoma Saos-2 Cells

**DOI:** 10.3390/ijms22083993

**Published:** 2021-04-13

**Authors:** Lukasz Bozycki, Joanna Mroczek, Laurence Bessueille, Saida Mebarek, René Buchet, Slawomir Pikula, Agnieszka Strzelecka-Kiliszek

**Affiliations:** 1 Laboratory of Biochemistry of Lipids, Nencki Institute of Experimental Biology, Polish Academy of Sciences, 3 Pasteur Str., 02-093 Warsaw, Poland; l.bozycki@nencki.gov.pl (L.B.); jgasik@chem.uw.edu.pl (J.M.); s.pikula@nencki.gov.pl (S.P.); 2 Department of Chemistry, University of Warsaw, 1 Pasteur Str., 02-093 Warsaw, Poland; 3 Department of Biosciences, Université de Lyon, CEDEX 69622 Villeurbanne, France; laurence.bessueille@univ-lyon1.fr (L.B.); saida.mebarek@univ-lyon1.fr (S.M.); rene.buchet@univ-lyon1.fr (R.B.); 4 Department of Biosciences, Université Lyon 1, CEDEX 69622 Villeurbanne, France; 5 INSA de Lyon, CEDEX 69621 Villeurbanne, France; 6 CPE Lyon, CEDEX 69616 Villeurbanne, France; 7 ICBMS CNRS UMR 5246, CEDEX 69622 Villeurbanne, France

**Keywords:** annexins, fetuin-A, mineralization, matrix vesicles, hFOB 1.19 osteoblastic cells, Saos-2 osteosarcoma cells

## Abstract

The mineralization process is initiated by osteoblasts and chondrocytes during intramembranous and endochondral ossifications, respectively. Both types of cells release matrix vesicles (MVs), which accumulate P_i_ and Ca^2+^ and form apatites in their lumen. Tissue non-specific alkaline phosphatase (TNAP), a mineralization marker, is highly enriched in MVs, in which it removes inorganic pyrophosphate (PP_i_), an inhibitor of apatite formation. MVs then bud from the microvilli of mature osteoblasts or hypertrophic chondrocytes and, thanks to the action of the acto-myosin cortex, become released to the extracellular matrix (ECM), where they bind to collagen fibers and propagate mineral growth. In this report, we compared the mineralization ability of human fetal osteoblastic cell line (hFOB 1.19 cells) with that of osteosarcoma cell line (Saos-2 cells). Both types of cells were able to mineralize in an osteogenic medium containing ascorbic acid and beta glycerophosphate. The composition of calcium and phosphate compounds in cytoplasmic vesicles was distinct from that in extracellular vesicles (mostly MVs) released after collagenase-digestion. Apatites were identified only in MVs derived from Saos-2 cells, while MVs from hFOB 1.19 cells contained amorphous calcium phosphate complexes. In addition, AnxA6 and AnxA2 (nucleators of mineralization) increased mineralization in the sub-membrane region in strongly mineralizing Saos-2 osteosarcoma, where they co-localized with TNAP, whereas in less mineralizing hFOB 1.19 osteoblasts, AnxA6, and AnxA2 co-localizations with TNAP were less visible in the membrane. We also observed a reduction in the level of fetuin-A (FetuA), an inhibitor of mineralization in ECM, following treatment with TNAP and Ca channels inhibitors, especially in osteosarcoma cells. Moreover, a fraction of FetuA was translocated from the cytoplasm towards the plasma membrane during the stimulation of Saos-2 cells, while this displacement was less pronounced in stimulated hFOB 19 cells. In summary, osteosarcoma Saos-2 cells had a better ability to mineralize than osteoblastic hFOB 1.19 cells. The formation of apatites was observed in Saos-2 cells, while only complexes of calcium and phosphate were identified in hFOB 1.19 cells. This was also evidenced by a more pronounced accumulation of AnxA2, AnxA6, FetuA in the plasma membrane, where they were partly co-localized with TNAP in Saos-2 cells, in comparison to hFOB 1.19 cells. This suggests that both activators (AnxA2, AnxA6) and inhibitors (FetuA) of mineralization were recruited to the membrane and co-localized with TNAP to take part in the process of mineralization.

## 1. Introduction

The mineralization process is initiated by osteoblasts and chondrocytes during intramembranous and endochondral ossification, respectively [1]. Both types of cells release matrix vesicles (MVs), which promote the accumulation of P_i_ and Ca^2+^, and form apatites in their lumen [2,3,4,5]. MVs, containing a considerable amount of tissue-nonspecific alkaline phosphatase (TNAP) and annexins [3], bud from the microvilli of osteoblasts or chondrocytes and are released to the extracellular matrix (ECM) [6,7,8,9,10]. Both annexins and TNAP have a collagen-binding capacity, which facilitates their accumulation along the collagen fibers to promote mineralization in the ECM scaffold [3,11]. TNAP hydrolyzes inorganic PP_i_, forming P_i_, while ectonucleotide pyrophosphatase/phosphodiesterase 1 (NPP1) hydrolyzes ATP producing PP_i_ and AMP. Both enzymes are the main regulators of the P_i_/PP_i_ ratio [3,12,13]. PP_i_ is an inhibitor of apatite formation [14]. A P_i_/PP_i_ ratio exceeding 142 is necessary to induce the formation of apatites [15].

Annexins are calcium- and phospholipid-binding proteins thought to participate in the influx of Ca^2+^ to MVs [5,16,17,18,19]. Annexins may bind to the actin cytoskeleton [20] and to S100 family proteins [21]. Several annexins, including AnxA6, AnxA5, AnxA2, and AnxA1, are present in MVs, which suggests that they have essential and possibly distinct functions during the mineralization process [22]. AnxA2 and AnxA6 are directly engaged in the formation of MVs in vascular smooth muscle cells (VSMC), thereby increasing their ability to mineralize [23]. AnxA5 was shown to initiate apatite nucleation at the inner side of MVs [24,25] and, when bound to the outer membrane of MVs, to interact with collagen, generating the mineralization process in both cases. Acidic pH-induced formation of AnxA6 ion channels [26]. A fraction of cellular Anx6 seems to be tightly bound or inserted into the bilayer of MV [27] since it could not be extracted by calcium chelators such as EGTA or EDTA [21]. AnxA6 present in MVs can be divided into three distinct pools. The first one corresponds to Ca^2+^-bound AnxA6 that interacts with the inner leaflet of the MV membrane. The second pool corresponds to AnxA6 localized on the surface of the outer leaflet. The third pool corresponds to AnxA6 inserted in the hydrophobic membrane bilayer and co-localized with cholesterol [27]. K201 (a benzodiazepine derivative), a potential inhibitor of annexin calcium channel activity, reduced the ability of MVs or extracellular vesicles from VSMCs to mineralize collagen fibers independently of whether it was added during or after MV formation by VSMC [23].

Fetuin-A (FetuA) prevents the growth and aggregation of minerals by forming FetuA-mineral complexes (CPP) [28]. Two types of such complexes can be distinguished:round, with disordered agglomerates of FetuA and minerals; longitudinal ones, with a mineral core and a protein shell formed by FetuA [29]. FetuA protein expression may prevent mineral bone disorder, cardiovascular disease, and chronic kidney disease [30,31,32]. FetuA uptake in bovine VSMCs is calcium-dependent and mediated by AnxA2 [33]. AnxA2 and AnxA6, expressed on the cell surface, were shown to serve as receptors for adhesion to immobilized FetuA [34] in breast carcinoma cells. TNAP also modifies mineral propagation by regulating the phosphorylation state of osteopontin (OPN), an inhibitor of mineral formation in ECM [8]. We hypothesized that interactions between members of the annexin family and FetuA should prevent osteoblast-mediated mineralization, similarly as in the case of interactions of FetuA with AnxA2 in VSMCs [33] as well as or with AnxA2 and AnxA6 in breast carcinoma cells [34]. To identify possible mechanisms and ways of preventing mineral bone disorders, we aimed to compared the mineralization competence of two human osteoblast-like cell lines: hFOB 1.19 osteoblasts (close to normal osteoblasts) and Saos-2 osteosarcoma (close to skeletal cancer) and to correlate the different examined intracellular distribution of AnxA2, AnxA6, FetuA, and TNAP in these cellular models with their mineralization competence.

## 2. Results

### 2.1. Characterization of the Mineralization Process in Human Fetal Osteoblastic Cell Line (hFOB 1.19 Cells) and Osteosarcoma Cell Lina (Saos-2 Cells)

**Figure 1:**
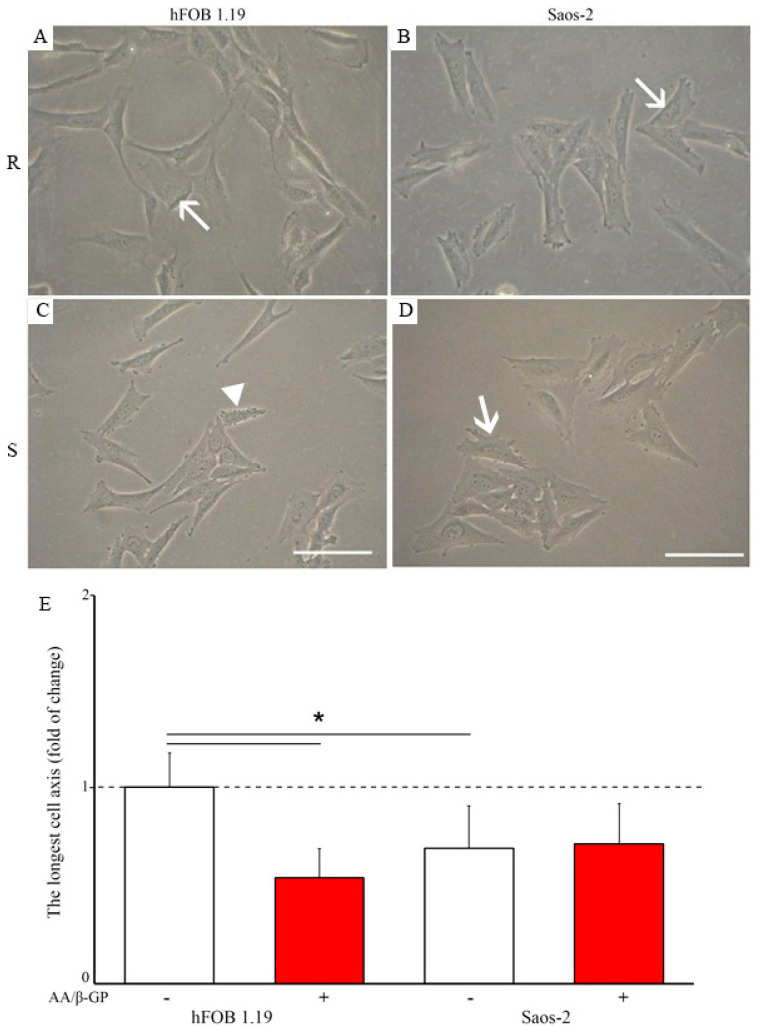
Morphology of hFOB 1.19 (**A**,**C**) and Saos-2 (**B**,**D**) cells in resting (**A**,**B**) and stimulated (**C**,**D**) conditions. Longest cell axis of cells in resting conditions (white) or after seven-day stimulation with ascorbic acid (AA) and β-glycerophosphate (β-GP) (red) observed under an Axiovert 40C light microscope (Carl Zeiss, Poznan, Poland) with Phase contrast, magnification at 120×. Arrows indicate an elongated cell morphology, whereas the arrowhead—a shortened cell axis. (**E**) In total, 500 cells were analyzed: 10 photographs were taken at each of five random locations of a 100 mm diameter dish, and the longest axis of 10 cells at each location was measured for each cell variant using Image J bundled with 64-bit Java 2.8.0_112 software (Bethesda, MD, USA) and presented as the fold of change of the longest axis of hFOB 1.19 resting cells; * *p* < 0.05.

**Figure 2:**
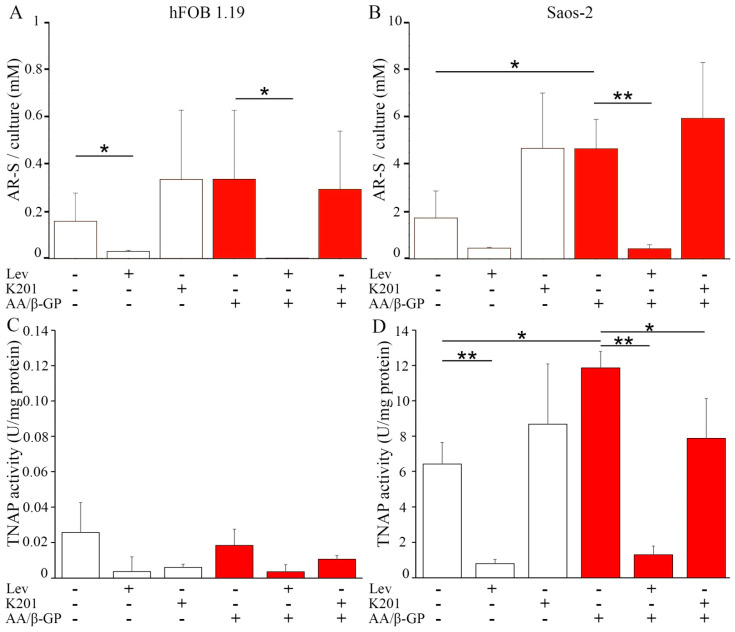
Mineralization level of hFOB 1.19 (**A**,**C**) and Saos-2 (**B**,**D**) cells in resting conditions (white) or after seven-day stimulation with AA and β-GP (red). Cells were either non-treated (−) or treated (+) with inhibitors: 100 μM levamisole (Lev) for Tissue non-specific alkaline phosphatase (TNAP) activity or 25 μM K201 for Ca channel activity. Ca salts (**A**,**B**) were stained with alizarin red-S (AR-S) dissolved in cetyl pyridinium chloride (CPC), and their content was measured spectrophotometrically at λ 562 nm. TNAP activity (**C**,**D**) was measured using alkaline phosphatase (ALP) Yellow para-nitro phenyl phosphate (pNPP) Liquid Substrate System for ELISA (Sigma-Aldrich, Warsaw, Poland), and absorbance was recorded spectrophotometrically at λ 405 nm. Data are means ± s.e. of at least three experiments; * *p* < 0.05 and ** *p* < 0.01.

Human fetal osteoblastic hFOB 1.19 and osteosarcoma Saos-2 cells were incubated for seven days in culture medium without (Resting, R) or with 50 µg/mL ascorbic acid (AA) and 7.5 mM β-glycerophosphate (β-G) (Stimulated, S). Under resting conditions, both types of cell had an elongated morphology (Figure 1A,B, arrows). The treatment with AA and β-GP induced, on average, a decrease in the longest axis of hFOB 1.19 cells by two-fold (Figure 1C, arrowhead, and Figure 1E), while resting and stimulated Saos-2 cells had a similar longest cell axis (Figure 1D, arrow, and Figure 1E). Quantitative alizarin red-S/cetyl pyridinium chloride (AR-S/CPC) analysis confirmed that stimulated cells produced more calcium nodules (hFOB 1.19—two times more, Saos-2—almost three times more) as compared with resting cells (Figure 2A,B, red histograms versus white histograms). The addition of levamisole (Lev) at 100 µM, a TNAP inhibitor, almost completely abolished the ability to produce calcium nodules in resting and stimulated hFOB 1.19 cells (Figure 2A) and Saos-2 cells (Figure 2B). Calcium nodules indicated the presence of calcium precipitates, but not necessarily the presence of apatite, as reported below. Inhibition of the activity of Ca channels by K201 seemed to induce more calcium nodules in hFOB 1.19 cells and in Saos-2 cells under resting conditions because resting cells (without stimulators like AA and β-GP) produce more amorphous calcium phosphate complexes, while it was without effect in stimulated hFOB 1.19 (Figure 2A) and Saos-2 (Figure 2B) cells maintained in conditions favoring conversion to apatite (with stimulators such as AA resulting in acidic pH and β-GP being a source of P_i_) [27]. TNAP activity in hFOB 1.19 osteoblastic cells was much lower than that in Saos-2 osteosarcoma cells, being almost on the threshold of detection (Figure 2C,D) [9]. Both stimulated hFOB 1.19, and Saos-2 cells had increased TNAP activity in comparison with resting cells (Figure 2C,D, red histograms versus white histograms). Treatment of the cells with Lev decreased the activity of TNAP to the basal level in both types of cells at both resting and stimulated conditions (Figs. 2C,D). The addition of K201 did not significantly affect TNAP activity in resting and stimulated hFOB cells (Figure 2C), whereas TNAP activity was slightly increased in resting Saos-2 cells and slightly decreased in stimulated Saos-2 cells compared with control cells (Figure 2D).

### 2.2. Analysis of Vesicular Fractions during Mineral Formation by Osteoblastic hFOB 1.19 and Osteosarcoma Saos-2 Cells

hFOB 1.19 and osteosarcoma Saos-2 cells were incubated for seven days under resting or stimulated conditions. They were treated with collagenase, and their extracellular media (ECM) were collected and subjected to differential centrifugation to obtain extracellular vesicles containing MVs. In the case of resting hFOB 1.19 cells, very small mineral particles were observed, as indicated by their black color in the fraction secreted to ECM (Figure S1A), while empty vesicles were observed in the fraction derived from the cytoplasm as evidenced by white vesicles on an uniform dark background (Figure S1B). Stimulation of hFOB 1.19 cells for mineralization resulted in the appearance in the cytoplasm of empty vesicles with increased diameter (Figure S1D, arrows). Electron-dense particles were observed in the ECM fraction released by hFOB 1.19 cells after stimulation, suggesting the presence of minerals inside MVs (Figure S1C, arrowheads). In the case of resting Saos-2 cells, aggregates of electron-dense, needle-shaped minerals were observed in the fraction derived from ECM (Figure S1E, arrowheads), whereas many empty small and middle-sized vesicles appeared in the cytoplasm (Figure S1F). Stimulation of Saos-2 cells for mineralization increased the number of vesicles and their diameter in the cytoplasm (Figure S1H, arrows), while electron-dense minerals were observed in ECM, suggesting the presence of mineral deposition and filled MVs (Figure S1G, arrowheads).

**Figure 3:**
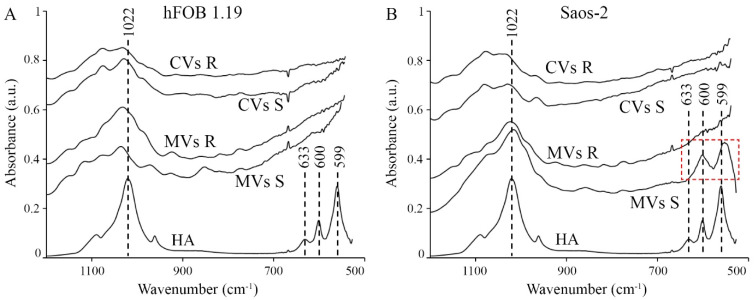
IR spectra of minerals formed by vesicular fractions of hFOB 1.19 (**A**) and Saos-2 (**B**) cells. Vesicular fractions were purified from the extracellular matrix (MVs) and from the cytoplasm (CVs) of collagenase-treated cells in resting conditions (R) or after stimulation with AA and β-GP (S). The vesicular fractions were dried. IR spectrum of apatite (HA) is characterized by peaks 1022, 633, 600, and 559 cm^−1^. The red rectangle indicates the presence of HA in the fraction of MVs derived from the Saos-2 cell line stimulated for mineralization. IR spectra were averaged from three independent samples, each measured at two distinct locations of the batch (*n* = 6). IR spectra were up scaled by 0.2 A to be better visible.

The vesicular fractions were analyzed for mineral composition by energy dispersive X-ray (EDX) microanalysis (Table 1) and IR spectroscopy (Figure 3). Ca/P ratios calculated from EDX measurements of TEM images indicate that, for both cell lines, values obtained in resting conditions were higher for the cytoplasmic samples (CVs) than for extracted ECM samples (MVs), whereas, after stimulating mineralization, they were higher for extracted ECM samples (MVs) than for cytoplasmic samples (CVs) (Table 1). Moreover, values of the Ca/P ratio for osteoblastic hFOB 1.19 cells did not exceed 1.0 and were lower, for all vesicle types and culture conditions, than the values obtained for osteosarcoma Saos-2 cells. In cancer cells, the values were higher than 1.0 for resting CVs and stimulated MVs, while a theoretical value of 1.67 is expected for hydroxyapatite (HA) [9]. IR spectrum for apatite (HA) (Figure 3A,B, bottom traces) was characterized by peaks corresponding to 1022, 633, 600, and 559 cm^−1^. Apatites were not observed, as indicated by the lack of 633, 600, and 599 cm^−1^ bands in the case of stimulated and resting hFOB 1.19 cells or in their ECM (MVsS and MVsR, stimulated and resting, respectively) and cytoplasmic (CVsS and CVsR, stimulated and resting, respectively) samples (Figure 3A). However, broad spectral features at around 1100–900 cm^−1^ may suggest the presence of calcium phosphate complexes and/or other unidentified materials. The band shapes and relative intensities are significantly different between samples from extracted ECM and cytoplasm, indicating distinct compositions of these complexes (Figure 3A, top traces (CVsS and CVsR) versus middle traces (MVsS and MVsR)). The presence of apatites was evidenced in the case of extracted ECM from Saos-2 cells under simulated conditions (Figure 3B, MVsS, red dashed square) since the spectrum possessed characteristic apatite bands, especially at 600 and 559 cm^−1^. The broad spectral feature at 1100–900 cm^−1^ may reveal various calcium complexes and/or other unidentified compounds that overlapped the 1022-cm^−1^ apatite band (Figure 3B, MVsS). The IR spectra of cytoplasm samples extracted from either hFOB 1.19 or Saos-2 stimulated and resting cells were similar (Figure 3A,B top traces (CVsS and CVsR). These spectra were significantly distinct from those of ECM samples (Figure 3A,B middle traces (MVsS and MVsR), suggesting that they contain distinct types of materials.

### 2.3. Protein Profiles of Mineralizing Osteoblastic hFOB 1.19 and Osteosarcoma Saos-2 Cells

Extracts of 5 × 10^8^ cells were homogenized in TLB buffer (as described in Section 4) and centrifuged. The pellets were analyzed by Western Blot to determine the protein content (Figure S2). We observed an increase in AnxA6 (70 kDa), AnxA2 (36 KDa), and FetuA (39 kDa) relative to actin (42 kDa) level after stimulation of hFOB 1.19 and Saos-2 cells. The level of AnxA6 was a little higher in Saos-2 cells t than in hFOB 1.19 cells (Figure S2A,C, white), whereas the level of AnxA2 was almost two times lower in Saos-2 than in hFOB 1.19 cells (Figure S2A,C, grey). FetuA content remained at a low level (below 1.0) in resting and stimulated hFOB 1.19 cells, whereas the level was high (above 1.0) in both resting and stimulated Saos-2 cells (Figure S2C, black). TNAP (55 kDa) remained at a middle level (around 1.0) in both types of cells, and its level did not change in stimulated conditions (Figure S2A, stripped).

After the addition of Lev, AnxA6, and AnxA2, levels were decreased in both examined cell lines (Figure S2A,B, white and grey, respectively). However, there was a pronounced decrease in the content of TNAP in stimulated versus resting Saos-2 cells treated with Lev, while no changes in TNAP were observed between resting and stimulated hFOB 1.19 cells as compared with control cells without inhibitor (Figure S2A,B, stripped).

We observed a significant decrease in AnxA6 and AnxA2 content upon the addition of K201 in resting and stimulated conditions in both examined cell lines (Figure S2C,D, white and grey, respectively). K201 diminished FetuA content in both resting and stimulated hFOB 1.19 and Saos-2 cells, in which the decrease was statistically significant (Figure S2C,D, black).

### 2.4. Protein Distributions in Resting and Stimulated hFOB 1.19 and Saos-2 Cells

**Figure 4:**
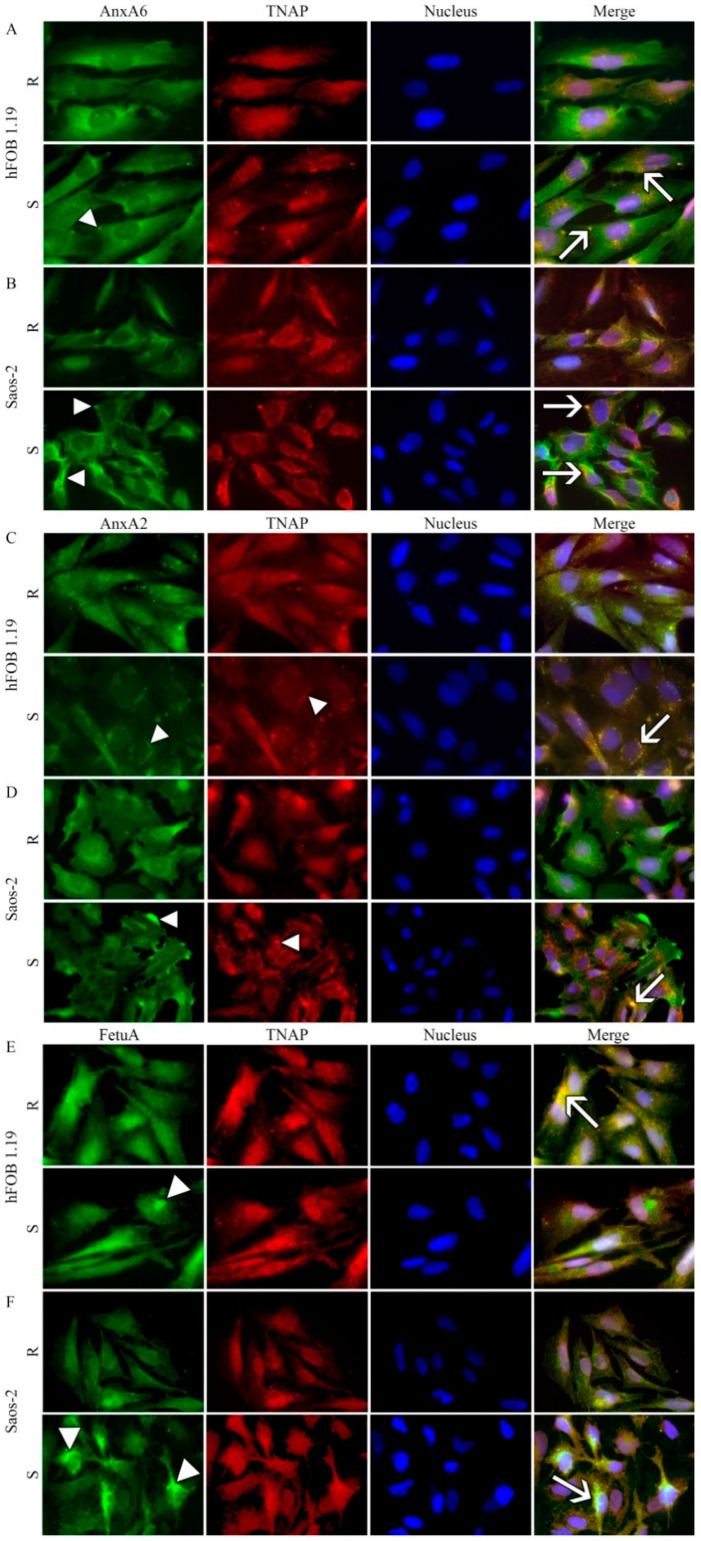
Co-localization of AnxA6, AnxA2, or fetuin-A (FetuA) with TNAP in hFOB 1.19 (**A**,**C**,**E**) and Saos-2 (**B**,**D**,**F**) cells in resting conditions (R) or after seven-day stimulation with AA and β-GP (S). The cells were incubated with appropriate antibodies: mouse monoclonal anti-AnxA6 (**A**,**B**), mouse monoclonal anti-AnxA2 (**C**,**D**), or mouse monoclonal anti-FetuA (**E**,**F**), all followed by goat anti-mouse IgG-FITC (green); rabbit polyclonal anti-TNAP followed by goat anti-rabbit IgG-TRITC (red) and DAPI for nuclei (blue) and observed under an Axio Observer.Z1 FM (Carl Zeiss, Poznan, Poland) with Phase contrast and appropriate fluorescent filters, magnification 630 x. Arrowheads indicate protein accumulation in vesicular and/or cluster structures. The yellow color and arrows on the merge images indicate AnxA6 (**A**,**B**), AnxA2 (**C**,**D**), or FetuA (**E**,**F**) co-localization with TNAP. Results of a typical experiment are presented.

In resting hFOB 1.19 (Figure 4A, upper panel) and Saos-2 (Figure 4B, upper panel) cells, AnxA6 was uniformly distributed. After stimulation of hFOB 1.19 cells for mineralization AnxA6 aggregated in the cytoplasm as indicated by bright spots (Figure 4A, lower panel, arrowhead), whereas in stimulated Saos-2 cells, it was accumulated at the inner surface of the cellular membrane as shown by extended spots along the membranes (Figure 4B, lower panel, arrowheads). In resting hFOB 1.19 (Figure 4A, upper panel), TNAP was also uniformly distributed, whereas in Saos-2 cells, a fraction of TNAP was co-localized with AnxA6 along the membrane (Figure 4B, upper panel, yellow color). Upon stimulation of hFOB 1.19 cells, a fraction of TNAP co-localized with AnxA6 aggregates (Figure 4A, lower panel, arrows), whereas in Saos-2 cells, it formed clusters as indicated by bright spots, which also co-localized with AnxA6 (Figure 4B, lower panel, arrows).

In resting hFOB 1.19 (Figure 4C, upper panel) and Saos-2 (Figure 4D, upper panel) cells, AnxA2 was uniformly distributed but in an aggregated form when compared to AnxA6 (Figure 4A,B). After stimulation of hFOB 1.19 cells for mineralization, cytoplasmic AnxA2 aggregates relocated to the perinuclear region as indicated by bright spots (Figure 4C, lower panel, arrowhead), whereas in stimulated Saos-2 cells, AnxA2 was accumulated at the surface of the cellular membrane as shown by extended spots along the membranes (Figure 4D, lower panel, arrowheads). In resting hFOB 1.19 (Figure 4C, upper panel), TNAP was also uniformly distributed, whereas in Saos-2 cells a fraction of total TNAP co-localized with AnxA2, as it did with AnxA6 (Figure 4A,B), along the membrane (Figure 4D, upper panel, yellow color). Upon stimulation of hFOB 1.19 cells, a pool of TNAP co-localized with AnxA2 aggregates (Figure 4C, lower panel, arrows), whereas in Saos-2 cells, it was forming clusters as indicated by bright spots which co-localized with AnxA2 (Figure 4D, lower panel, arrows) similarly as with AnxA6 (Figure 4A,B).

In resting hFOB 1.19 cells, FetuA accumulated in the cytoplasm in the perinuclear region (Figure 4E, upper panel), whereas in Saos-2 cells, it was uniformly distributed in the cytoplasm (Figure 4F, upper panel). After stimulation of hFOB 1.19 cells for mineralization, FetuA remained in the cytoplasm but appeared to be more aggregated (Figure 4E, lower panel, arrowhead), whereas, in stimulated Saos-2 cells, FetuA redistributed to the sub-membrane and perinuclear regions (Figure 4F, lower panel, arrowheads). In resting hFOB 1.19 (Figure 4E, upper panel), as well as in Saos-2 cells, a pool of TNAP co-localized with FetuA (Figure 4E,F), similarly as with AnxA6 (Figure 4A,B) and AnxA2 (Figure 4C,D), along the membrane (Figure 4E,F, upper panels, yellow color). Upon stimulation of hFOB 1.19 cells, a pool of TNAP co-localized with FetuA aggregates (Figure 4E, lower panel, arrow), whereas in Saos-2 cells, it formed clusters as indicated by bright spots, which also co-localized with FetuA (Figure 4F, lower panel, arrows) similarly as with AnxA6 (Figure 4B) and AnxA2 (Figure 4D).

**Figure 5:**
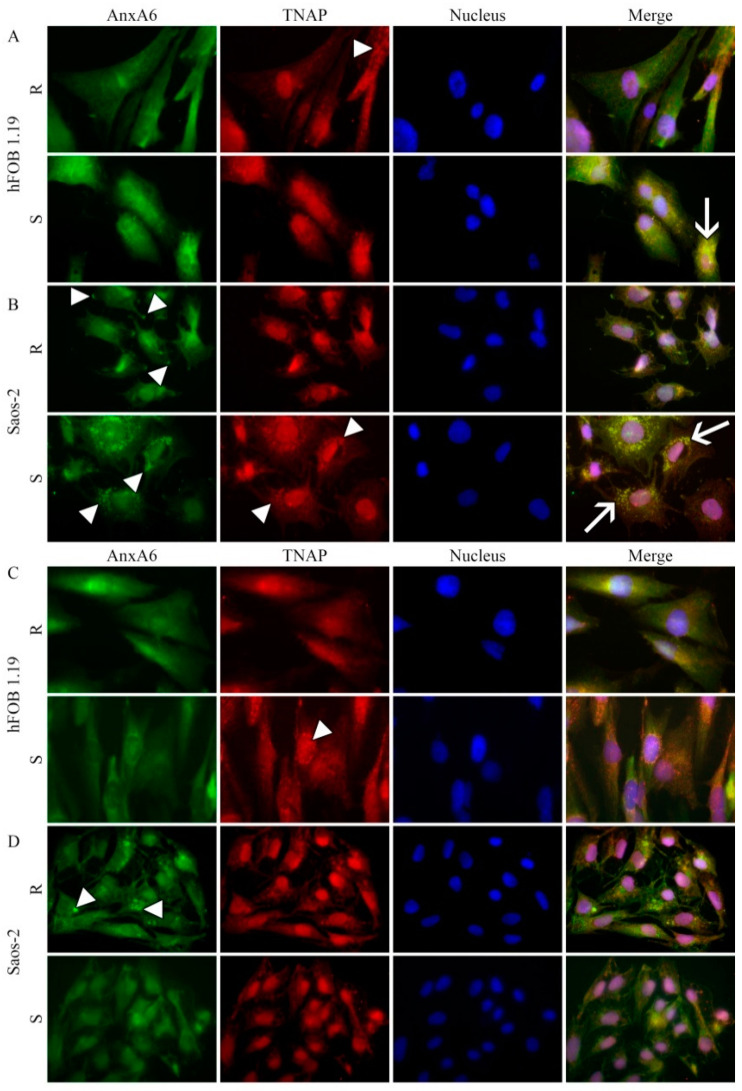
Co-localization of AnxA6 with TNAP in hFOB 1.19 (**A**,**C**) and Saos-2 (**B**,**D**) cells after treatment with 100 μM levamisole (**A**,**B**) or 25 μM K201 (**C**,**D**) in resting conditions (R) or after seven-day stimulation with AA and β-GP (S). The cells were incubated with appropriate antibodies: mouse monoclonal anti-AnxA6 followed by goat anti-mouse IgG-FITC (green), rabbit polyclonal anti-TNAP followed by goat anti-rabbit IgG-TRITC (red) and DAPI for nucleus (blue) and observed under an Axio Observer.Z1 FM (Carl Zeiss, Poznan, Poland) with Phase contrast and appropriate fluorescent filters, magnification at 630×. Arrowheads indicate protein accumulation in vesicular and/or cluster structures. The yellow color and arrows on the merge images indicate AnxA6 co-localization with TNAP. Results of a typical experiment are presented.

Inhibition of TNAP activity by Lev altered cell morphologies toward a round shape, and the distribution of AnxA6 and TNAP appeared less aggregated (Figure 5A,B) as compared to cells without Lev treatment (Figure 4). Blocking the activity of calcium channels by K201 slightly affected the distribution of AnxA6 and TNAP. In both cells, lines AnxA6 and TNAP were less co-localized and did not form clusters upon stimulation (Figure 5C,D) as compared with cells without K201 treatment (Figure 4).

In resting hFOB 1.19 cells, FetuA accumulated in the cytoplasm in the perinuclear region (Figure S3A, upper panel, arrowhead), whereas in Saos-2 cells, it was uniformly distributed in the cytoplasm (Figure S3B, upper panel). After stimulation of hFOB 1.19 cells for mineralization, FetuA remained in the cytoplasm, but a fraction of the protein was translocated to lamellipodia (Figure S3A, lower panel, arrowhead). In stimulated Saos-2 cells, FetuA was redistributed to the sub-membrane region (Figure S3B, lower panel, arrowheads). In resting hFOB 1.19 cells, β-actin was in the form of fibers, which were as long as the length of the cell (Figure S3A, upper panel), whereas, in Saos-2 cells, it formed fibers beneath the plasma membrane and evenly distributed throughout the cell (Figure S3B, upper panel). In stimulated hFOB 1.19 cells, the amount of actin fibers increased in regions close to the membrane (Figure S3A, lower panel, arrowhead), and they co-localized with FetuA (Figure S3A, lower panel, arrows). In stimulated Saos-2 cells, β-actin was visible as short fibers uniformly distributed in the whole-cell, and some granular forms relocated to membrane regions (Figure S3B, lower panel). The absence of yellow color on merge images suggested that β-actin did not co-localize with FetuA (Figure S3B, lower panel, arrows).

The addition of Lev influenced cell morphology, making cells round, and thus interfered with FetuA and β-actin distributions because the structure of stress fibers was not preserved. In resting hFOB 1.19 cells, FetuA accumulated in the perinuclear region (Figure S3C, upper panel, arrowhead), whereas in resting Saos-2 cells, apart from the perinuclear region, it accumulated in the endings of membrane appendages and a pool of FetuA coincided with β-actin localization as indicated by yellow color (Figure S3D, upper panel, arrowheads) that were more pronounced in stimulated cells (Figure S3D, lower panel, arrowhead). In stimulated hFOB 1.19 cells, the addition of the inhibitor dispersed FetuA in the whole cell (Figure S3C, lower panel). In resting cells, β-actin was visible in the form of densely arranged fibers that formed the sub-membrane cytoskeleton of both types of cells. In stimulated cells, it was clearly visible that β-actin translocated toward membrane regions, and this was confirmed by its tight co-localization with FetuA (Figure S3C,D, lower panels, arrows).

The presence of K201 affected the morphology of both cell types either under resting and stimulated conditions (Figure S3E,F) as compared with cells without the addition of K201 (Figure S3A,B). We noted a significant increase in the intensity of the FetuA signal, probably because it accumulated in membranous structures in stimulated hFOB 1.19 cells (Figure S3E, lower panel, arrowhead) and in resting Saos-2 cells (Figure S3F, upper panel, arrowhead). The structure of β-actin fibers was impaired mainly in osteosarcoma Saos-2 cells (Figure S3F) as compared with Saos-2 cells without K201 (Figure S3B). The redistribution of β-actin toward the plasma membrane and co-localization with FetuA occurred mostly in stimulated cells (Figure S3E,F, lower panels, arrows).

Relative co-localization areas calculated from coefficient measurements of FM-ApoTome images indicate that, for both cell lines, values were obtained for all tested proteins (AnxA6, AnxA2, and FetuA vs. TNAP or β-actin) after stimulation for mineralization were almost twice as higher than in resting conditions (Table 2). Treatment of cell cultures with Lev increased the relative co-localization areas between AnxA6/TNAP and FetuA/β-actin, mainly in hFOB 1.19 resting and stimulated cells. The addition of K201 to the cell cultures increased the relative co-localization areas between AnxA6/TNAP, mainly in resting Saos-2 cells, and FetuA/β-actin mainly in stimulated Saos-2 cells (Table 2).

## 3. Discussion

In this report we compared the mineralization ability of osteoblastic hFOB 1.19 cells with that of osteosarcoma Saos-2 cells. hFOB 1.19 cells adhere to the surface irregularly and mineralize at the apical part of the membrane, whereas Saos-2 cells attach to the surface regularly and mineralize along the whole membrane surface, including lamellipodia (Figure 1). Osteosarcoma Saos-2 cells mineralized around 10 times more efficiently than osteoblastic hFOB 1.19 cells (Figure 2A,B), probably due to almost 100 times higher activity of TNAP (Figure 2C,D) as was previously reported [6,7,9]. The hydrolysis of PP_i_ to P_i_, the main TNAP function in mineralizing cells, seems to be more crucial for the mineralization process than the transport of Ca^2+^ by annexins or by other unidentified channels. This is evident from the results on cell-induced mineralization after the addition of TNAP inhibitor (Figure 2) or the addition of K201, a calcium transport blocker. We demonstrated that stimulation for mineralization increased the number and size of the vesicles, which in turn facilitated the formation of bigger minerals in the ECM, especially in the case of Saos-2 cells (Figure S1). We identified for the first time the differences between the two types of vesicular structures observed in the cells. We found that MVs are smaller and filled with minerals (have dense particles inside), whereas CVs are bigger and empty, with few mineral deposited on their walls (do not contain dense particles), and basically resemble multivesicular bodies (MVBs) (Figure S1C versus Figure S1H). hFOB 1.19 osteoblasts induced the formation of amorphous calcium phosphate complexes that probably need more time to be converted to apatites. Minerals from Saos-2 cells were composed of mixtures of amorphous calcium phosphate complexes and apatites with Ca/*p*-ratios approaching to the theoretical value for HA (Figure 3, Table 1). Our findings confirm findings from recent data based on the empirical phenomenon of the primary mineralization process via MV-mediated mechanism and the transformation which these structures undergo during bio-mineralization [35].

Stimulation of cells for mineralization enhanced the expression level of AnxA2, mainly in hFOB 1.19 osteoblasts, and of AnxA6, mainly in Saos-2 cells, which is consistent with increased mineralization ability of both cell lines upon stimulation. FetuA level increased in Saos-2 cells almost two times comparing to hFOB 1.19 cells. Treatment of both cells with Lev slightly decreased TNAP and AnxA6 levels in Saos-2 cells, whereas AnxA2 decreased in hFOB 1.19 cells (Figure S2A,B). Treatment of cells with K201 strongly decreased FetuA and AnxA6 levels in Saos-2 cells, whereas AnxA2 decreased in hFOB 1.19 cells (Figure S2C,D). Stimulation for mineralization increased the concentration of both annexins, AnxA6 (Figure 4A,B) and AnxA2 (Figure 4C,D), in the sub-membrane region and their co-localization with TNAP in strongly mineralizing Saos-2 osteosarcoma cells (Table 2). In hFOB 1.19, osteoblasts, which mineralized less, AnxA6 and AnxA2 co-localization with TNAP in the sub-membrane region were less visible (Table 2). Upon stimulation, a fraction of FetuA aggregates co-localized with TNAP in hFOB 1.19 cells, whereas in Saos-2 cells, both FetuA and TNAP formed clusters that were strongly co-localized (Figure 4E,F, Table 2). Treatment of both cell lines with Lev (Figure 5A,B) or with K201 (Figure 5C,D) shifted AnxA6 localization toward the membrane and altered its co-localization with TNAP. Our results from bone cells are in agreement with data showing that in retinoic acid (RA)-treated growth plate chondrocytes, K201 significantly attenuated the expression of terminal differentiation marker genes, such as cbfa1, TNAP, OCN, and type I collagen. Furthermore, K201 inhibited the up-regulation of AnxA2, 5, and 6 gene expressions in these cells. RA-treated chondrocytes released mineralization-competent MVs, which contained significantly higher amounts of AnxA2, 5, and 6, as well as TNAP activity than vesicles isolated from untreated or RA/K-201-treated cultures. Consistently, only RA-treated cultures showed significant mineralization [16,36]. On the other hand, lack of AnxA6 resulted in reduced TNAP activity and Ca^2+^ and P_i_ content in MVs, and in the inability to form apatite-like crystals in vitro [37].

Cells stimulated for mineralization start to round up because stress fibers are shortened. FetuA was accumulating at the membranous regions in mineralizing Saos-2 cells. This observation is consistent with an earlier report for AnxA6 showing that β-actin did not co-localize with FetuA in mineralizing osteosarcoma cells [10]. However, in hFOB 1.19 osteoblasts, which mineralize less, FetuA still co-localized with β-actin in focal contacts (Figure S3A,B). In both cell lines, the treatment with Lev changed FetuA localization in that FetuA was strongly co-localized with β-actin, especially in stimulated Saos-2 cells (Figure S3C,D). On the other hand, the addition of K201 to the cell cultures did not induce co-localization of FetuA with β-actin (Figure S3E,F). Our results are comparable with previous data presenting that accumulation of Ca^2+^ and PO_4_^3-^ inside MVs initiates crystalline nucleation associated with the inner leaflet of MVs. Calcium phosphate crystals elongate radially, penetrate MVs membrane, and finally grow out of the vesicles to form calcifying nodules, that is, globular assemblies of needle-shaped mineral crystals retaining some of the transporters and enzymes such as TNAP and AnxA5. The subsequent growth of calcifying nodules appears to be regulated by the surrounding organic compounds, such as FetuA, finally leading to collagen mineralization [38].

To conclude, as reported previously, osteosarcoma Saos-2 cells had a better ability to mineralize as osteoblastic hFOB 1.19 cells [9]. Our findings suggest that membranous co-localization of several activators of apatite formation, such as TNAP and AnxA6, and inhibitors of apatite formation, such as FetuA, is a necessary prerequisite for controlled and balanced mineralization.

## 4. Materials and Methods

### 4.1. Cell Culture and Treatment

Human fetal hFOB 1.19 SV40 large T antigen transfected osteoblastic cells (ATCC CRL-11372, LGC Standards, PL) were cultured in a 1:1 mixture of Ham’s F12 medium and Dulbecco’s modified Eagle’s medium with 2.5 mM L-glutamine (ATCC, LGS Standards, PL) supplemented with 100 U/mL penicillin, 100 U/mL streptomycin (Sigma-Aldrich, Warsaw, Poland), 0.3 mg/mL G418 (Sigma-Aldrich, Warsaw, Poland) and 10% Fetal Bovine Serum (*v/v*, FBS, Gibco, Thermo Fisher Scientific, Warsaw, Poland). The cells were grown at 34 °C in the atmosphere of 5% CO_2_.

Human osteosarcoma Saos-2 cells (ATCC HTB-85, LGC Standards, Warsaw, Poland) were cultured in McCoy’s 5A medium with 1.5 mM L-glutamine (ATCC, LGC Standards, Warsaw, Poland) supplemented with 100 U/mL penicillin, 100 U/mL streptomycin (Sigma-Aldrich, Warsaw, Poland) and 15% FBS (*v/v*, Gibco, Thermo Fisher Scientific, Warsaw, Poland). The cells were grown at 37 °C in the atmosphere of 5% CO_2_.

Saos-2 and hFOB 1.19 cells were stimulated for mineralization one day after cell passage and attachment by treatment with 50 μg/mL ascorbic acid (AA, Sigma-Aldrich, Warsaw, Poland) and 7.5 mM β-glycerophosphate (β-GP, Sigma-Aldrich, Warsaw, Poland) for 7 days [39]. Cell cultures were treated (i) without any further additions; (ii) with the addition of 100 μM, (S)-(−)-6-Phenyl-2,3,5,6-tetrahydroimidazo[2,1-b]thiazolehydrochloride, L(−)-2,3,5,6-Tetrahydro-6-phenylimidazo[2,1-b]thiazole hydrochloride, (Lev, levamisole, an inhibitor of TNAP, Sigma-Aldrich, Warsaw, Poland) or iii) with 25 μM 1-(2,3-Dihydro-7-methoxy-1,4-benzothiazepin-4(5H)-yl)-3-[4-(phenylmethyl)-1-piperidinyl]-1-propanone hemifumarate (K201, JTV-519, an inhibitor of Ca channel activity, Sigma-Aldrich, Warsaw, Poland) for 7 days starting 4 h after the addition of AA and β-GP. The final concentration of DMSO, as a solvent for K201 solutions, in the culture medium did not exceed 0.1% (*v*/*v*). Cell cultures were observed under an inverted Axiovert 40C light microscope (Carl Zeiss, Poznan, Poland) with Phase contrast. In total, 500 cells were analyzed: 10 photographs were taken at each of 5 random locations of a 100 mm diameter dish, and the longest axis of 10 cells at each location was measured for each cell variant using Image J bundled with 64-bit Java 2.8.0_112 software (Bethesda, MD, USA).

### 4.2. Calcium Minerals Detection

The whole procedure of alizarin red-S (AR-S) staining followed by cetyl pyridinium chloride (CPC) de-staining of calcium deposits was performed as described earlier [10].

### 4.3. Collagenase-Treatment, Vesicular Fractions, and Electron Microscopy with EDX

Cells numbering 10^8^, either resting or stimulated for 7 days, were digested according to a collagenase digestion protocol [23]. Medium from cell cultures was collected while cells were washed with phosphate-buffered saline (PBS) and incubated with crude collagenase (500 U/mL, type IA; Sigma-Aldrich, Warsaw, Poland) in a solution of 0.25 M sucrose, 0.12 M NaCl, 0.01 M KCl, and 0.02 M Tris-HCl buffer, pH 7.45, at 37 °C for 3 h. Then, cells were mechanically scraped, passed 10 times through a 0.5 × 16 syringe, sonicated twice on ice for 10 s at 20% power of an S-250D digital sonifier (Branson Ultrasonic S.A., Merck, Warsaw, Poland) and centrifuged at 800× *g* for 5 min at 4 °C (MPW-350R, MPW Medical Instrument, Warsaw, Poland) to remove cell debris and then at 30,000× *g* to remove microsomes. The supernatant was centrifuged at 250,000× *g* to collect vesicles from the cytoplasm (CVs). The pellet was suspended in 500 μL of Hank’s balanced salt solution (HBSS, 5.4 mM KCl, 0.3 mM Na_2_HPO_4_, 0.6 mM KH_2_PO_4_, 0.6 mM MgSO_4_, 137 mM NaCl, 5.6 mM D-glucose, 2.38 mM NaHCO_3_, pH 7.4).

The collected medium, after collagenase digestion, was centrifuged at 1000× *g* to remove cell debris and then at 100,000× *g* to collect the fraction of vesicles secreted by the cells, including vesicles of the extracellular matrix (MVs) and other types of extracellular vesicles (EVs).

Ten microliters of vesicular fractions (CVs and MVs) were negatively stained as follows: placed on Formavar/Carbon 300 mesh Ni grids (Agar Scientific, Stansted, UK), incubated at 20 °C for 30 min, stained in 2.5% uranyl acetate in 50% ethanol in darkness at 20 °C for 20 min, washed once in 50% ethanol and three times in deionized water and dried at 20 °C for 24 h. Then, samples were observed under a JEM 1400-TEM (Jeol Co., Tokyo, Japan) electron microscope equipped with an INCA energy dispersive X-ray microanalysis (EDX) system (Oxford Instruments, Oxfordshire, UK) and an 11 Mega pixel MORADA G2 camera (Olympus Soft Imaging Solutions, Tokyo, Japan). The spectral and compositional analyses were carried out on point measurements of elements by the INCA software in TEM images, and the Ca/P ratios were calculated for each sample, as described previously [9].

### 4.4. IR Spectra of Minerals Formed by Vesicular Fractions

The pellets, obtained after collagenase digestion followed by ultracentrifugation (as described in Section 4.3), were dried at −50 °C with 500 mBar pressure using an Alpha 1–2 Freeze Dryer (Christ & Co., Berlin, Germany). Dried pellets were deposited on an attenuated total reflectance plate to analyze mineral composition using a Nicolet 510M Infrared spectrometer (Nicolet, Thermo Fisher Scientific, Lyon, France) equipped with a deuterated triglycine sulfate detector [15]. Sixty-four interferograms were recorded at a 4 cm^−1^ optical resolution. The presented spectra were averaged from 6 spectra collected from 3 independent probes. Each independent probe was taken from two distinct locations of the batch to verify the homogeneity of the batch.

### 4.5. Cell Lysis and TNAP Activity Assay

10^8^ cells, either resting or stimulated for 7 days, were lysed in TLB (0.1% Triton X-100, 0.1% β-mercaptoethanol, 1 mM EDTA, 1 mM EGTA, 1 µg/mL Protease Inhibitor Cocktail (Sigma-Aldrich, Warsaw, Poland), 0.2 mM PMSF, 2 mM NaF, 2 mM Na_3_VO_4_, 50 mM Tris-HCl, pH 8.0) buffer. Medium from cell cultures was removed, while cells were washed with PBS and incubated with 1 mL of lysis buffer at 4 °C for 15 min. Then, cells were mechanically scraped, vortexed for 10 s, sonicated on ice for 10 s at 20% power of an S-250D digital sonifier (Branson Ultrasonic S.A., Merck, Warsaw, Poland), and centrifuged at 800× *g* for 10 min at 4 °C (MPW-350R, MPW Medical Instrument, Warsaw, Poland). The collected supernatant was analyzed for protein concentration using the Micro BCA Reagent (Pierce, Merck, Warsaw, Poland), and absorbance was measured at 562 nm in a BioMate3 spectrophotometer (Thermo Electron Co., Waltham, MA, USA). The same supernatant was also analyzed for TNAP activity using the alkaline phosphatase (ALP) Yellow pNPP (para-nitro phenyl phosphate) Liquid Substrate System for ELISA (Sigma-Aldrich, Warsaw, Poland) as described earlier [10]. The reaction was initiated by the addition of 10 μL (0.5 μg of protein) aliquots of the supernatant fraction to 96-well plates containing 200 μL of pNPP as substrate. The plates were preincubated at 37 °C for 5 min, and absorbance was recorded at 405 nm for 1 h with 15 s intervals using a Spectra Max M5e multi-detection reader (Molecular Devices). The reaction was stopped using 50 μL of 3 M NaOH. TNAP activity was calculated as U/mg protein, where 1 U = 1 μmol pNPP hydrolyzed per min and visualized by the Origin 7.5 software (Origin Co., Electronic Arts, Los Angeles, CA, USA).

### 4.6. SDS-PAGE and Immunoblot Analysis

Proteins of cell lysates were separated on 10% (*w/v*) SDS-PAGE [40] and then electro-transferred (Mini-ProteanIITM Kit, Bio-Rad, Hercules, CA, USA) onto nitrocellulose membranes (HybondTM-ECLTM, Amersham Biosciences, GE Healthcare, Little Chalfont, UK) according to Towbin et al. [41]. Nitrocellulose membranes were blocked with 5% (*w/v*) milk in TBS for 1 h at room temperature. The membranes were then incubated with mouse monoclonal anti-annexin A6 (AnxA6; 1:1000, *v/v*; BD Transduction Laboratories, Warsaw, Poland), mouse monoclonal anti-annexin A2 (AnxA2; 1:1000, *v/v*; BD Transduction Laboratories, Warsaw, Poland), mouse monoclonal anti-fetuin-A (FetuA; 1:500, *v/v*; Abcam, Cambridge, UK), rabbit polyclonal anti-TNAP (TNAP; 1:1000, *v/v*; Abcam, Cambridge, UK), or mouse monoclonal anti-actin 1 (Actin; 1:2000, *v/v*; Abcam, Cambridge, UK) primary antibodies prepared in 3% (*w/v*) milk in TBS supplemented with 0.05% (*v/v*) Tween-20 (TTBS), at 4 °C overnight. Nitrocellulose membranes were washed several times with TTBS and then incubated for 2 h at room temperature with sheep anti-mouse IgG secondary antibody conjugated with horseradish peroxidase (1:5000, *v/v*; Amersham Biosciences, GE Healthcare, Little Chalfont, UK) and prepared in 3% (*w/v*) milk in TTBS. Finally, the membranes were washed, and immunoreactive bands were visualized on MXB X-ray films (Kodak) using ECL reagents according to the manufacturer’s instructions (Amersham Biosciences, GE Healthcare, Little Chalfont, UK). Then the films were analyzed densitometricaly using the InGenius software (Syngene, Cambridge, UK).

### 4.7. Immunochemistry and Fluorescent Microscopy with ApoTome

Cells numbering 10^5^ were cultured in culture medium on cover slips overnight at 37 °C in 5% CO_2_ humidified atmosphere. The next day, the stimulators (50 μg/mL AA and 7.5 mM β-GP) and, 4 h later, inhibitors (either 100 μM Lev, or 25 μM K201), were added to the appropriate cell culture variants for 7 days. Then cells were washed with PD buffer (125 mM NaCl, 5 mM KCl, 10 mM NaHCO_3_, 1 mM KH_2_PO_4_, 10 mM glucose, 20 mM HEPES, and pH 6.9) and fixed with 3% (*w/v*) paraformaldehyde in PD buffer (20 min, room temperature) [6]. Fixed cells were incubated in 50 mM NH_4_Cl in PD buffer (10 min, room temperature), washed with PD buffer, and then permeabilized with 0.08% (*v/v*) Triton X-100 in PD buffer (5 min, 4 °C). After additional washing with Tris-buffered saline (TBS; 100 mM NaCl, 10 mM Tris-HCl, and pH 7.5), cells were incubated with a blocking solution, 5% (*v/v*) FBS in TBS, for 45 min at room temperature. Then, cells were incubated with mouse monoclonal anti-annexin A6 (AnxA6; 1:100, *v/v*; BD Transduction Laboratories, Warsaw, Poland), mouse monoclonal anti-annexin A2 (AnxA2; 1:100, *v/v*; BD Transduction Laboratories, Warsaw, Poland), mouse monoclonal anti-fetuin-A (FetuA; 1:100, *v/v*; Abcam, Cambridge, UK), or rabbit polyclonal anti-TNAP (TNAP; 1:100, *v/v*; Abcam, Cambridge, UK) primary antibodies diluted in TBS containing 0.5% FBS (*v/v*) and 0.05% Tween-20 (*v/v*). After 1 h of incubation at room temperature, the cells were washed in TBS and incubated for 1 h at room temperature with goat anti-mouse IgG-fluorescein isothiocyanate (FITC; 1:200, *v/v*; Sigma-Aldrich, Warsaw, Poland) or goat anti-rabbit IgG-tetramethylrhodamine isothiocyanate (TRITC; 1:200, *v/v*; Sigma-Aldrich, Warsaw, Poland) secondary antibodies or with Phalloidin-TexasRed from *Amonita phalloides* (F-actin; 1:1500, *v/v*; Fluka, Sigma-Aldrich, Warsaw, Poland) diluted in TBS containing 0.5% FBS (*v/v*) and 0.05% Tween-20 (*v/v*). After washing, with TBS and deionized water, the samples were mounted in 0.6% (*v/v*) Moviol 4–88 (Calbiochem, Merck, Warsaw, Poland) supplemented with 2.5% (*w/v*) DABCO (Sigma-Aldrich, Warsaw, Poland). Samples were stored at 4 °C overnight and then photographed under an Axio Observer.Z1 fluorescent microscope (Carl Zeiss, Poznan, Poland) equipped with an ApoTome optical sectioning in fluorescence imaging to remove out-of-focus light using a patented algorithm for structured illumination. The co-localization coefficients were measured by Axio Vision Rel. 4.8 software (Carl Zeiss, Poznan, Poland) in FM images for each channel. The extent of co-localization was calculated by summing the pixels in the co-localized region (Quadrant 3) and then dividing by the sum of pixels either in Channel 1 (Quadrant 1 + 3) or in Channel 2 (Quadrant 2 + 3) to indicate the relative co-localization area in percent for each sample. Each pixel had a value of 1.

### 4.8. Statistical Analysis

All values are reported as mean ± sd. Data were analyzed by one-way ANOVA, and post hoc analyses were performed using the Tukey method with the aid of Daniel’s XL Toolbox add-in for Excel, version 6.60, by Daniel Kraus, Wurzburg, GE [42]. Statistical significance was described as * *p* < 0.05, ** *p* < 0.01, and *** *p* < 0.001.

## Figures and Tables

**Table 1:** Ca/P ratio calculated for vesicular fractions derived from extracellular matrix (mostly MVs) and cytoplasm (CVs) of collagenase-treated hFOB 1.19 or Saos-2 cells in resting conditions (R) or after stimulation with AA and β-GP (S). Data are means ± s.e., *n* = 8–18 measurements of three independent experiments (* *p* < 0.05, ** *p* < 0.01, and *** *p* < 0.001).

	Vesicle Type	Ca/P Ratio			
		hFOB 1.19 Cells		Saos-2 Cells	
**R**	CVs	0.59 ± 0.17	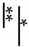	0.92 ± 0.57	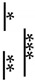
	MVs	0.11 ± 0.06		0.19 ± 0.03	
**S**	CVs	0.15 ± 0.06		0.31 ± 0.09	
	MVs	0.28 ± 0.25		1.06 ± 0.22	

**Table 2:** **Relative co-localization area** calculated for AnxA6, AnxA2 or FetuA vs. TNAP or β-actin in hFOB 1.19 or Saos-2 cells in resting conditions (R) or after stimulation with AA and β-GP (S). Data are means ± s.e., *n* = 3 measurements of three independent experiments (* *p* < 0.05 and ** *p* < 0.01).

	Inhibitor Type	Protein Type	Relative Co-Localization Area (%)			
			hFOB 1.19 Cells		Saos-2 Cells	
**R**	-	AnxA6/TNAP	12.93 ± 1.49	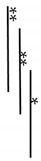	12.60 ± 0.88	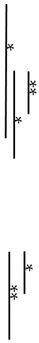
		AnxA2/TNAP	10.86 ± 1.55		16.59 ± 1.20	
		FetuA/TNAP	12.51 ± 2.11		16.61 ± 1.84	
		FetuA/β-actin	13.40 ± 1.79		9.03 ± 0.43	
	Lev	AnxA6/TNAP	31.72 ± 3.65		17.93 ± 0.83	
		FetuA/β-actin	33.33 ± 4.61		14.69 ± 2.28	
	K201	AnxA6/TNAP	19.46 ± 1.39		24.37 ± 4.26	
		FetuA/β-actin	20.87 ± 0.68		12.82 ± 0.67	
**S**	-	AnxA6/TNAP	17.40 ± 1.21		22.54 ± 1.49	
		AnxA2/TNAP	16.03 ± 2.33		21.98 ± 5.09	
		FetuA/TNAP	17.79 ± 0.46		22.23 ± 3.78	
		FetuA/β-actin	18.24 ± 0.79		11.90 ± 1.53	
	Lev	AnxA6/TNAP	24.27 ± 3.43		21.57 ± 1.54	
		FetuA/β-actin	21.68 ± 0.20		19.23 ± 0.69	
	K201	AnxA6/TNAP	16.02 ± 0.96		18.39 ± 1.31	
		FetuA/β-actin	14.59 ± 2.87		23.25 ± 4.90	

## Data Availability

Not applicable.

## Supplementary Materials

The following are available online at https://www.mdpi.com/article/10.3390/ijms22083993/s1, Figure S1: Vesicular fractions in hFOB 1.19 and Saos-2 cells, Figure S2: Presence of TNAP, FetuA, AnxA6 and AnxA2 level in hFOB 1.19 and Saos-2 cells, as determined by Western Blot, Figure S3: Co-localization of FetuA with β–actin in hFOB 1.19 and Saos-2 cells after treatment with 100 μM levamisole or 25 μM K201.

## Abbreviations

AA: ascorbic acid; ALP, alkaline phosphatase; AnxA2, AnxA6, vertebrate annexins A2, A6; AR-S, Alizarin Red-S; β-GP, β-glycerophosphate; CPC, cetyl pyridinium chloride; CVs, cellular vesicles; ECM, extracellular matrix; FetuA, fetuin-A; K201, benzodiazepine derivative; Lev, levamisole; MVs, matrix vesicles; P_i_, inorganic phosphate; pNPP, para-nitro phenyl phosphate; PP_i_, inorganic pyro phosphate; TNAP, tissue non-specific alkaline phosphatase.
