# Supplementary material for: Annexins A2, A6 and Fetuin-A Affect the Process of Mineralization in Vesicles Derived from Human Osteoblastic hFOB 1.19 and Osteosarcoma Saos-2 Cells
Source: Int J Mol Sci. 2021 Apr 13;22(8):3993. doi: 10.3390/ijms22083993 (PMC8069967; doi:10.3390/ijms22083993)
Supplement: Supplementary file 1 [file ijms-22-03993-s001.pdf]

Supplementary Figure legends

Figure S1. Vesicular fractions in hFOB 1.19 (A-D) and Saos-2 (E-H) cells derived from extracellular matrix (mostly MVs) (A, C, E, G) and cytoplasm (CVs) (B, D, F, H) in resting conditions (R) or after stimulation with AA and β-GP (S). Arrowheads indicate electron dense material, arrows – empty vesicles. Fractions were obtained by collagenase digestion followed by ultracentrifugation, probes were observed under JEM 1400-TEM (Jeol Co., Tokyo, Japan), magnification 50 000 x, bar = 500nm.

Figure S2. Presence of TNAP, FetuA, AnxA6 and AnxA2 level in hFOB 1.19 and Saos-2 cells, as determined by Western Blot. Cells were either non-treated (-) or treated (+) with 100 μM Lev (A, B) or 25 μM K201 (C, D), in resting conditions (-) or after 7-day stimulation with AA and β-GP (+). Whole cell lysates were prepared in TLB. WB were incubated with appropriate primary (rabbit anti-TNAP, mouse anti-FetuA, mouse anti-AnxA6, mouse anti-AnxA2, mouse anti-ActinI) followed by secondary (sheep anti-rabbit or anti-mouse IgG-HRP) antibodies. The level of presented proteins was quantified using the InGenius software (Syngene, Cambridge, UK), calculated per actin level and presented as the fold of change of the protein content for TNAP (A, stripped), FetuA (C, black), AnxA6 (A and C, white), AnxA2 (A and C, grey), n=3-4, *p<0.05, **p<0.01. The sample of hFOB 1.19 cells in resting conditions, for which TNAP to actin level ratio was taken as 1, was a reference sample. Representative WB after Lev (B) or K201 (D) are also presented.

Figure S3. Co-localization of FetuA with β-actin in hFOB 1.19 (A, C) and Saos-2 (B, D) cells (A, B) after treatment with 100 μM levamisole (C, D) or 25 μM K201 (E, F) in resting conditions (R) or after 7-day stimulation with AA and β-GP (S). The cells were incubated with appropriate antibodies: mouse monoclonal anti-FetuA followed by goat anti-mouse IgG-FITC (green) and phalloidin-Rhodamine from *Amanita phalloides* (red) and DAPI for nucleus (blue) and observed under an Axio Observer.Z1 fluorescent microscope (Carl Zeiss, Poznan, PL) with phase contrast and appropriate fluorescent filters, magnification 630 x. Arrowheads indicate protein accumulation in vesicular and/or cluster structures. The yellow color and arrows on the merge images indicate FetuA co-localization with β-actin. Data of a typical experiment are presented.

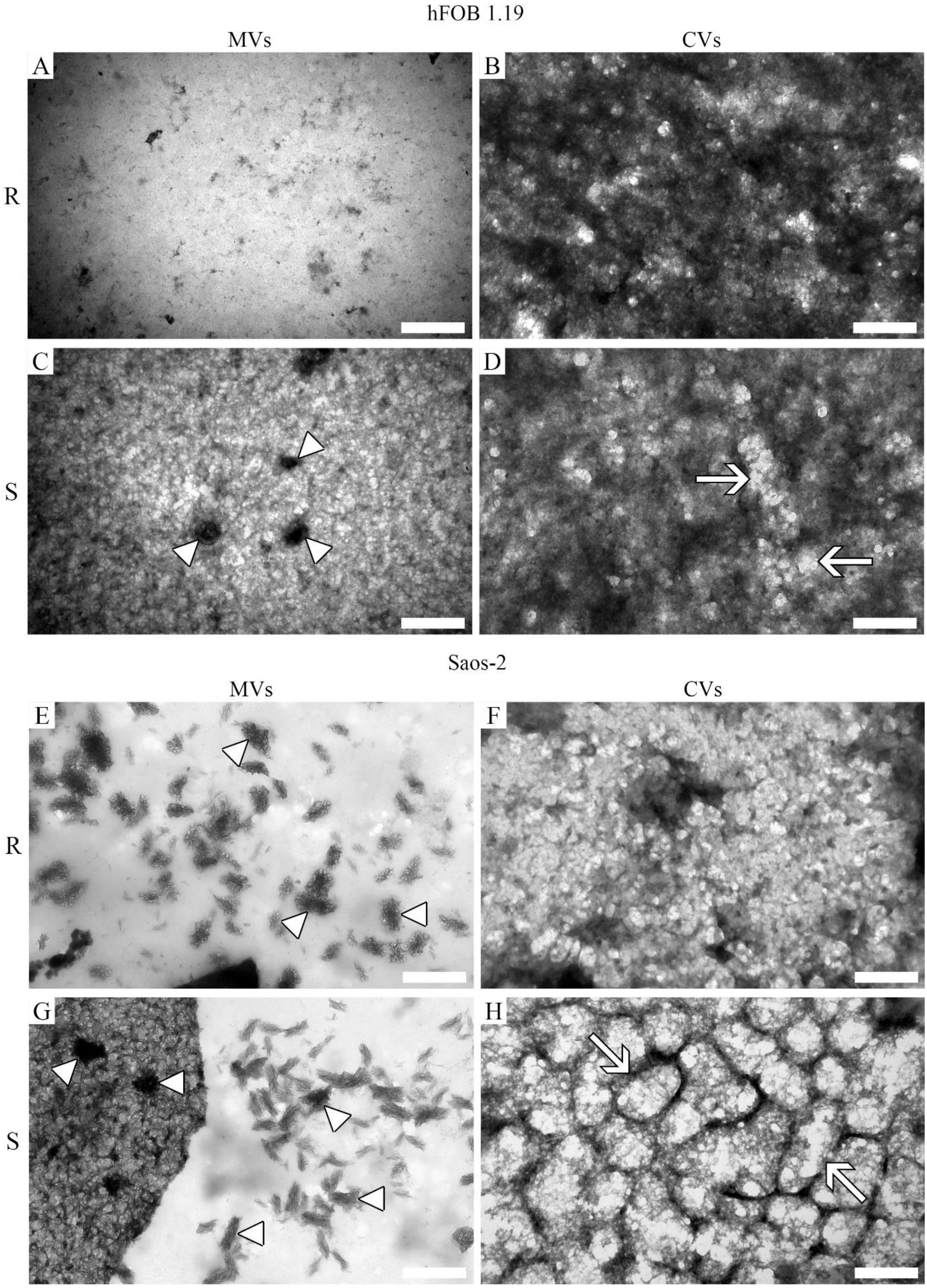

Figure S1.

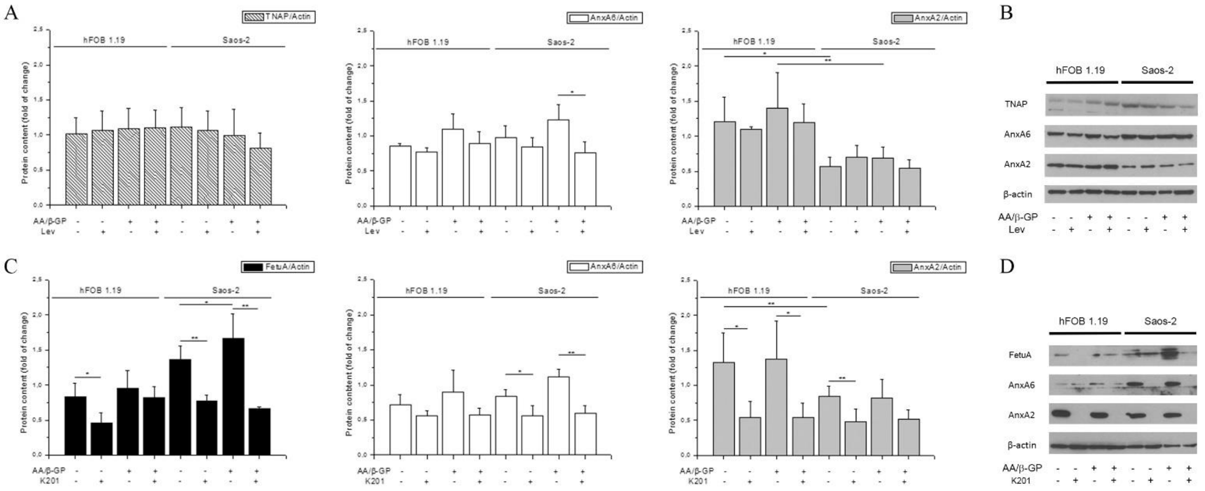
A
TNAP/Actin
AnxA6/Actin
AnxA2/Actin
hFOB 1.19
Saos-2
Protein content (fold of change)
AA/β-GP
Lev
B
hFOB 1.19
Saos-2
TNAP
AnxA6
AnxA2
β-actin
AA/β-GP
Lev
C
FetuA/Actin
AnxA6/Actin
AnxA2/Actin
hFOB 1.19
Saos-2
Protein content (fold of change)
AA/β-GP
K201
D
hFOB 1.19
Saos-2
FetuA
AnxA6
AnxA2
β-actin
AA/β-GP
K201

Figure S2.

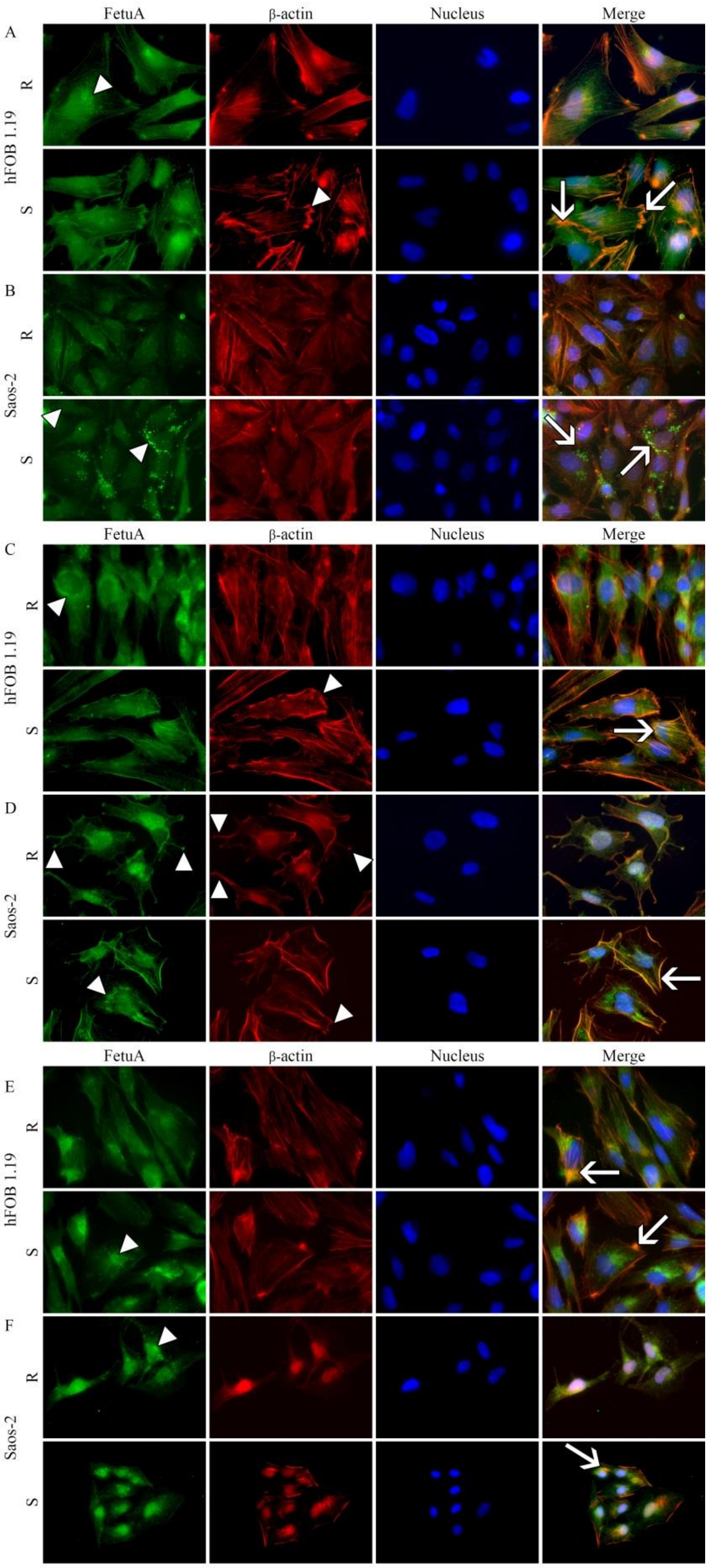
FetuA
β-actin
Nucleus
Merge
A
hFOB 1.19
R
S
B
Saos-2
R
S
FetuA
β-actin
Nucleus
Merge
C
hFOB 1.19
R
S
D
Saos-2
R
S
FetuA
β-actin
Nucleus
Merge
E
hFOB 1.19
R
S
F
Saos-2
R
S

Figure S3.
